# A Model for the Training Effects in Swimming Demonstrates a Strong Relationship between Parasympathetic Activity, Performance and Index of Fatigue

**DOI:** 10.1371/journal.pone.0052636

**Published:** 2012-12-20

**Authors:** Sébastien Chalencon, Thierry Busso, Jean-René Lacour, Martin Garet, Vincent Pichot, Philippe Connes, Charles Philip Gabel, Frédéric Roche, Jean Claude Barthélémy

**Affiliations:** 1 Laboratory EA4607 SNA-EPIS, Jean Monnet University of Saint-Etienne, PRES Lyon, Saint-Etienne, France; 2 Laboratory of Exercise Physiology EA4338, Jean Monnet University of Saint-Etienne, PRES Lyon, Saint-Etienne, France; 3 UMR Inserm 665, Ricou Hospital, Academic Hospital of Pointe a Pitre, Pointe-à-Pitre, Guadeloupe; 4 University of the Sunshine Coast, Queensland, Australia; University of Adelaide, Australia

## Abstract

Competitive swimming as a physical activity results in changes to the activity level of the autonomic nervous system (ANS). However, the precise relationship between ANS activity, fatigue and sports performance remains contentious. To address this problem and build a model to support a consistent relationship, data were gathered from national and regional swimmers during two 30 consecutive-week training periods. Nocturnal ANS activity was measured weekly and quantified through wavelet transform analysis of the recorded heart rate variability. Performance was then measured through a subsequent morning 400 meters freestyle time-trial. A model was proposed where indices of fatigue were computed using Banister’s two antagonistic component model of fatigue and adaptation applied to both the ANS activity and the performance. This demonstrated that a logarithmic relationship existed between performance and ANS activity for each subject. There was a high degree of model fit between the measured and calculated performance (R^2^ = 0.84±0.14,p<0.01) and the measured and calculated High Frequency (HF) power of the ANS activity (R^2^ = 0.79±0.07, p<0.01). During the taper periods, improvements in measured performance and measured HF were strongly related. In the model, variations in performance were related to significant reductions in the level of ‘Negative Influences’ rather than increases in ‘Positive Influences’. Furthermore, the delay needed to return to the initial performance level was highly correlated to the delay required to return to the initial HF power level (p<0.01). The delay required to reach peak performance was highly correlated to the delay required to reach the maximal level of HF power (p = 0.02). Building the ANS/performance identity of a subject, including the time to peak HF, may help predict the maximal performance that could be obtained at a given time.

## Introduction

In order to achieve the best performance in sports, training follows several basic laws that include the relationship between training intensity and recovery. The successive bouts of intensive training and recovery have specific criteria. Modeling can be performed to optimize sporting performances and prevent excessive fatigue which may impede recovery. This has been proposed [1] as a two antagonistic component model of fatigue and adaptation. The autonomic nervous system (ANS) activity has been described as a marker correlated to performance, measured in terms of VO_2max_ [2] and its variations can be related to a single bout of exercise [3], however age may be a confounding factor. From a longitudinal point of view, ANS activity has been related to training and recovery periods for both teams [4] and individuals [5]. Furthermore, it has been highlighted that the ANS activity during training must be accounted for [6]. This specific aspect of training has demonstrated the necessity to avoid low ANS activity periods as this may limit the potential benefits of training.

Training programs use specific combinations of intensity, volume and frequency [7] repeated over consecutive sessions to reach a given level of performance. The goal remains to optimize performance at a key period [8], [9] through the use of alternations in intensive training (IT) periods which may determine overreaching states [10], [11] as well as in taper recovery (TR) periods. The optimized combination of these two aspects will determine the athletes’ highest performance capacity [4], [12].

While it is not yet possible to build a full-season training program, several mathematical tools have been proposed to describe the effects of physical training on performance [13]–[16]. More importantly, these tools improve our understanding of the effects of successive IT and TR periods [16]–[22]. However, these studies have not used ANS activity as an integrative quantified parameter.

Technically, the characteristics of training load for these two periods of IT and TR need to be taken into account [1]. They must also be related to performance via a transfer function that includes two first-order filters. These can account for the difference between the two components: first, that acting positively on performance and training adaptations; and second, that acting negatively, specifically the level of fatigue [23]. This technique requires a high level of data density from consecutive days and weeks in order to examine the quantitative relationship between the amount of work performed and the improvement in physical performance.

Within this context we considered that the ANS activity may represent a putative parameter that could be modeled against performance. Furthermore, the data and indices are easily recorded and calculated allowing possible practical applications. The goal would be to assess if the variations in ANS activity occurring with training can be modeled to provide useful indices of the balance between fatigue and adaptation. This would provide a powerful analytical tool for training performance as well as optimizing individual health. This last benefit would be due to the ANS activity level being an optimal health marker [24].

The aims of the study were to: 1) compare the response of performance and ANS activity to training; 2) to build a model to support a consistent relationship between ANS activity, fatigue and sports performance.

## Methods

Using available mathematical methods for ANS activity measurements [25] we followed ten skilled swimmers over thirty consecutive weeks that included two periods of intensive training and recovery. This enabled the provision of a model that considered the possible balances between performance and ANS activity indices. By using data from the individuals’ response to training, the proposed model may help provide insights towards achieving optimal training effects and subsequent performance.

### Subjects

Ten swimmers of regional to national level were recruited into the study: six males (age: 17.3±2.8) and four females (age: 15.8±1.9 years). The participants had a mean intensive training history of 5.6±2 years and mean training duration of 10.7±2.9 hours per week during the season. All were injury free and did not take medication during the period of the study. Written informed consent was obtained from either the subjects or their parents in the case of minors. The study was approved by the Jean Monnet University and University North hospital ethics committee.

### Experimental Method

Two consecutive cycles that included IT and TR were monitored over thirty consecutive training weeks. The first cycle lasted 15 weeks and consisted of progressive increases and decreases in training load. It included an intensive four week training period (weeks 9–12) and a three week taper period (weeks 13–15). The second cycle immediately followed, and lasted 16 weeks. It included an intensive five week training period (weeks 22–26) and a four week recovery period (weeks 27–30). Each week the training load was quantified. The ANS activity was recorded during sleep on the Thursday night and performance was assessed on the Friday morning by a 400-meter freestyle time-trial. The day before these measurements, subjects were asked to avoid caffeine and alcohol intake.

### Nocturnal ANS Activity Assessment (Appendix S1)

Nocturnal recordings were performed using a Polar S-810 monitor, skin-connected through complementary cables and electrodes to avoid recording discontinuity. The inter-beat (RR) intervals were visually validated then Wavelet Transform was applied to extract the components of heart rate variability (refer to Appendix S1). Wavelet Transform was selected in preference to Fourier Transform as it allows the analysis of non-stationary signals and greater precision in ANS variable assessment with no additional computing CPU time [25].

The variables determined from the Wavelet analysis were: High Frequency (HF), which represented the parasympathetic; Low Frequency (LF), for the sympathetic; the ratio of Low/High frequencies (LF/HF), for the equilibrium; and Total Power Frequency (Ptot) variations, for the sum of ANS activity [25].

Variables brought by the time domain analysis were calculated which included: the percentage of differences between adjacent normal RR intervals >50 ms (PNN50); the standard deviation of all normal RR intervals (SDNN); the square root of the mean of the sum of the squared differences between adjacent normal RR intervals (RMSSD); the standard deviations of the mean of all normal RR intervals for 5-minute segments (SDANN); and the mean of the standard deviation of all normal RR intervals for all 5 minutes segments (SDNNIDX).

### Quantification of the Systems Input and Output (Appendix S2)

The application of the systems model used in this study requires a precise longitudinal quantification of the daily training amount. This relates training (systems input) to performance and nocturnal ANS activity (systems output). The systems input domain of training amount was considered as the sum of the number of pool-kilometers swum or the dry land workout equivalent weighted by a specific coefficient according to seven training intensities (refer to Appendix S2). In order to avoid the variations that may be related to a disruption in the training intensity, the exercise sessions preceding the HRV and performance measurements were very accurately controlled. The two systems output domains were defined with respect to specific criteria as follows: The criterion of performance was a value for the mean velocity during the 400-meter time-trial transformed to a percentage of the best-performed national value. Because previous studies suggested that parasympathetic activity and sports performance might be related [4]–[6], the HF spectral component of the HRV was the criterion for ANS activity.

### Mathematical Modeling of the Responses to Training (Appendix S3)

Measurements were undertaken to establish a dose-response relationship based on the positive and negative influences of training on the systems output. The model, originally built to analyze the variations in performance, was based on the two opposite effects of training: the positive effect ascribed to adaptation; and the negative effect ascribed to fatigue [1]. The systems output is the balance between these two components. The positive effect is a slow adaptation process and remains relatively steady within the period after each training bout. The negative effect varies more rapidly, presenting first a deep negative effect on the output then being rapidly corrected as the adaptation process dominates. As a consequence, a greater increase in the negative than the positive component provoked a transient decrease in output during intensified training periods. A subsequent reduction in training amount allowed the negative component to dissipate more quickly than the positive, yielding an output peak. The parameters of this mathematical relationship, two gain terms (k_1_ and k_2_) and two time constants (τ_1_ and τ_2_) for the positive and negative component respectively, characterize the dynamic behavior of the output (performance or ANS activity) in response to a training bout.

The modeling of the responses to physical training requires several steps. First, the model parameters were determined by fitting modeled output to measured data by the least square method. This procedure is applied to measured and estimated performance and also to HF power values. Second, the estimates of k_1_, k_2_, τ_1_ and τ_2_ obtained in the first step were used to calculate the time to recover the initial level of performance and HF power after a training bout (t_n_P_ and t_n_HF_, respectively), and the time to reach peak performance and peak HF power (t_g_P_, and t_g_HF_, respectively). Third, adaptation and fatigue indices are computed from the positive and negative components of the model [15]. The values of positive influence and negative influence on performance (PI_P and NI_P) and on HF power (PI_HF and NI_HF) were calculated each day from the sum of influences of each previous training amount.

### Statistics

The normality of distribution was tested for each participant using the Shapiro-Wilk test (p<0.05). Consequently Performance, HF power, negative and positive influences of training were compared using two-way repeated-measures factorial ANOVA with the Bonferroni procedure as the post-hoc test and the parametric T-Student test was used as determined by the variables normality of distribution. The effect of gender was evaluated for all variables studied using an unpaired t-test. As no significant gender difference was observed all data variables were pooled for a single analysis. Pearson’s correlation coefficient was used to study the relationship between the modeled parameters for performance and HF power.

## Results

### Fitting of Performance to HF Values

**Figure 1:**
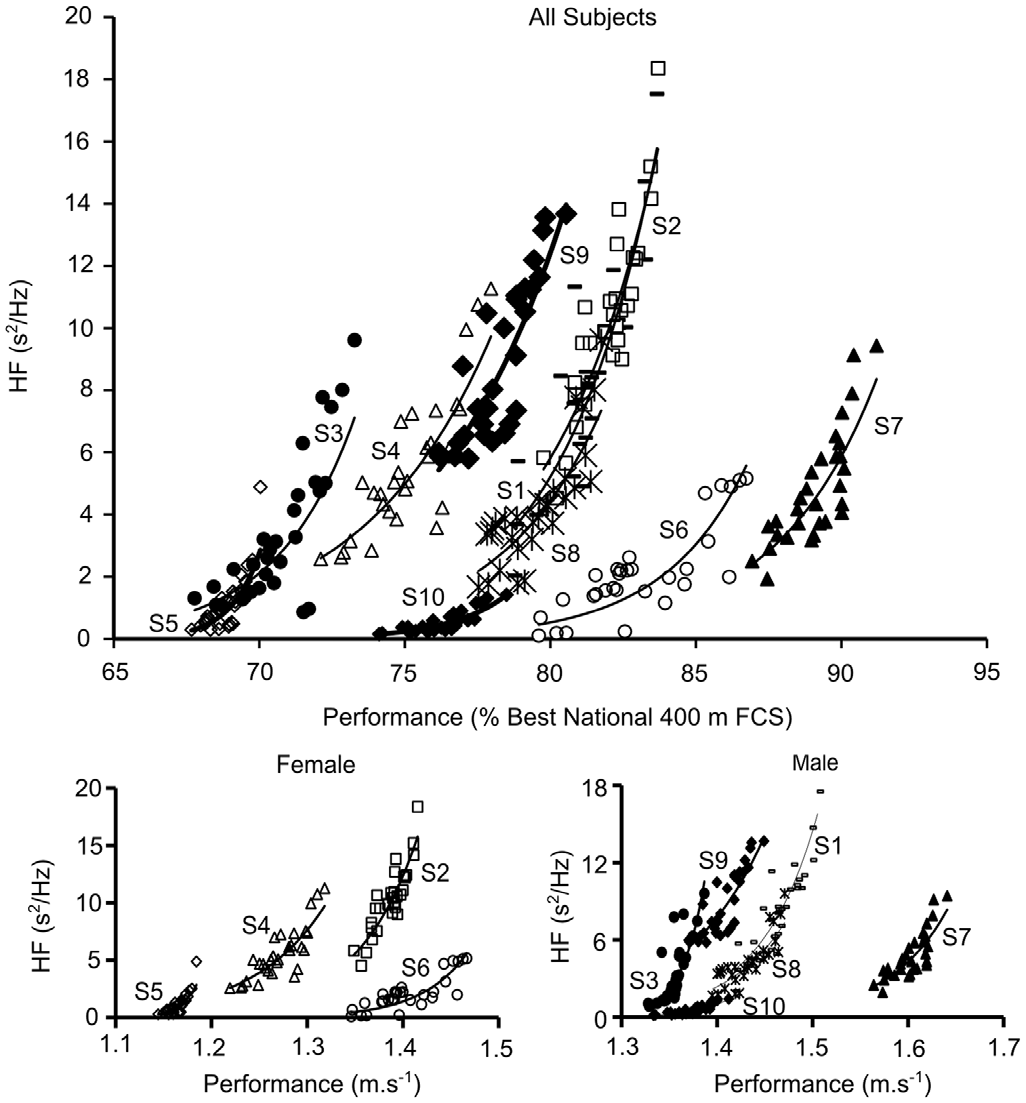
Relationship between HF (s^2^/Hz) and Performance (% best national Performance). Representation for four female (S2, S4, S5, S6) and six male subjects (S1, S3, S7, S8, S9, S10). Best performances were: Female: 1.42 m.s^−1^ (S2), 1.32 m.s^−1^ (S4), 1.18 m.s^−1^ (S5) and 1.47 m.s^−1^ (S6); and Male: 1.51 m.s^−1^ (S1), 1.39 m.s^−1^ (S3), 1.64 m.s^−1^ (S7), 1.47 m.s^−1^ (S8), 1.45 m.s^−1^ (S9) and 1.41 m.s^−1^ (S10).

Best raw performances were: for men - 1.51 m.s^−1^ (S1), 1.39 m.s^−1^ (S3), 1.64 m.s^−1^ (S7), 1.47 m.s^−1^ (S8), 1.45 m.s^−1^ (S9) and 1.41 m.s^−1^ (S10); for women - 1.42 m.s^−1^ (S2), 1.32 m.s^−1^ (S4), 1.18 m.s^−1^ (S5) and 1.47 m.s^−1^ (S6). Performances were then transformed to percentages of the best performed national value (Figure 1).

When individual HF data were plotted against individual performance data, a highly significant relationship appeared for each of the ten subjects (p<0.001). The R^2^ values ranged from 0.55 to 0.80 (S1 0.76, S2 0.80, S3 0.69, S4 0.71, S5 0.63, S6 0.55, S7 0.69, S8 0.65, S9 0.72, and S10 0.73) (Figure 1). Increased HF power values were associated with higher performance. Others ANS indices did not correlate with performance, neither did others wavelet indices or temporal indices of heart rate variability. The lowest performance (S5) showed the smallest performance increase with its change in HF power value. However, it was not possible to establish a significant relationship between the mean slope of the fitting curves and the performances.

### Estimation of Performances and HF Values Fitting

**Figure 2:**
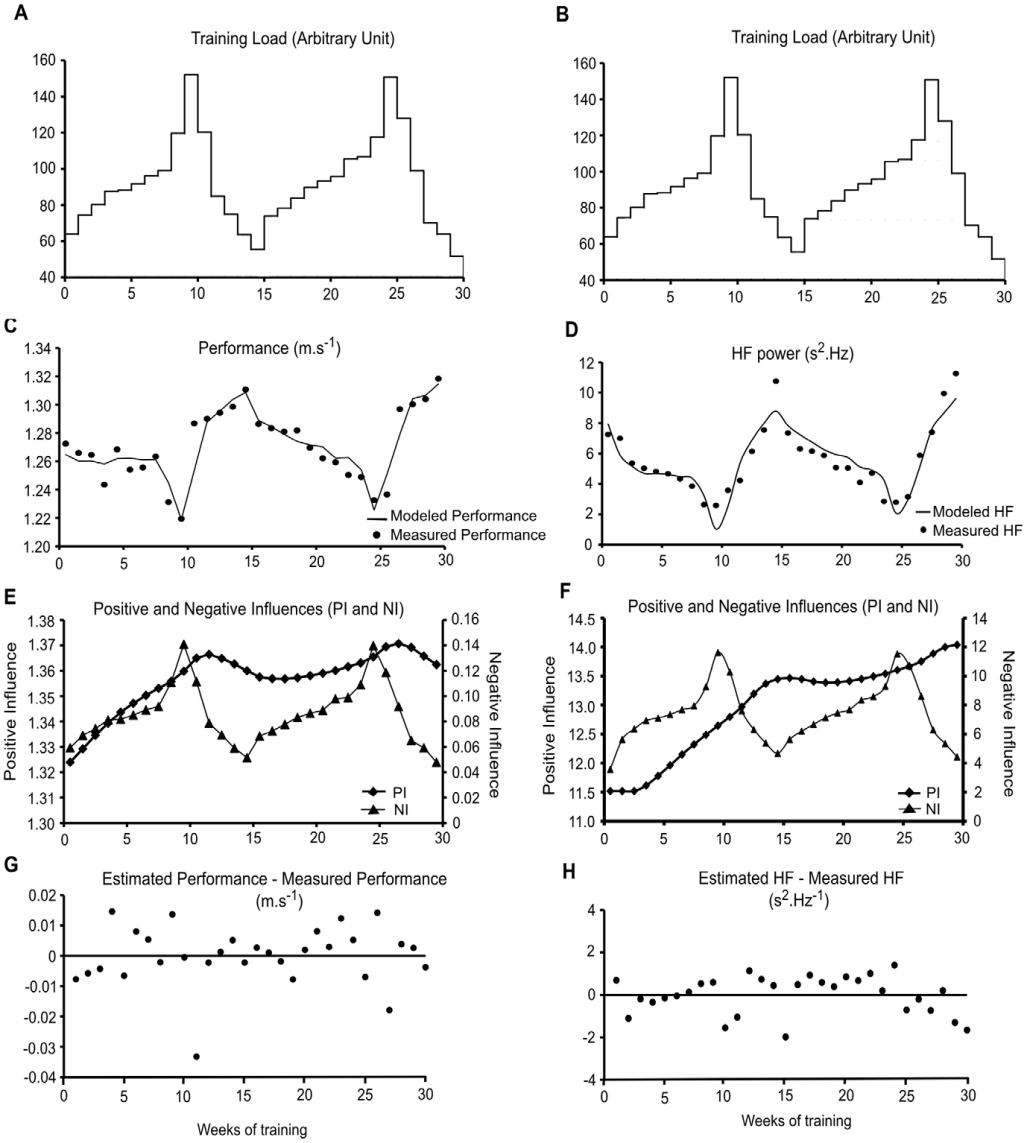
Application of the two-component model to swimmer #4. The left column is for Performance, the right column for HF Power. Row 1 panels (A) and (B) represent the same daily amount of training per week as a reference for interpreting lower panels. Row 2 panels respectively represent the fit between modeled and measured performance (C) and HF (D) in response to training. Row 3 panels represent profiles of positive and negative influences on performance (E) and on HF power (F), expressed in the same units as (C) and (D). Row 4 panels represent the residual difference between measured and estimated performance (G) and HF power (H).

The measured performances (Figure 2, Panel B, left side, dots) demonstrated a sharp decrease during the two intensive training periods (Figure 2, Panel A) and a sharp reincrease responding to the decrease in training intensity. This continued until more intense training resumed. A similar observation was found for the HF values (Figure 2, Panel B, right side, dots). During the two training cycles, the model demonstrated a high fit between measured and estimated performance and between measured and estimated HF values (Figure 2, Panel C and D, respectively). The residuals between measured and estimated values are low, as illustrated by the high R^2^ values (Table 1). However, we observed some individual measured HF data points slightly farther from the modeled curve, particularly peak HF values during both recovery periods, at the rebound (Figure 2, Panel C and D, respectively).

**Table 1:** Indicators of goodness-of-fit (R^2^ values) between calculated and measured Performance and between modelled and measured HF power.

	S1	S2	S3	S4	S5	S6	S7	S8	S9	S10	mean
**Performance**	0.84	0.91	0.83	0.92	0.91	0.86	0.90	0.89	0.45	0.86	0.84±0.14
**HF power**	0.78	0.77	0.77	0.84	0.68	0.69	0.86	0.84	0.85	0.78	0.79±0.07

The model fitting was also evaluated for other Wavelet Transform variables, particularly LF (sympathetic activity), Ptot (the total energy) and the LF/HR ratio (the equilibrium between sympathetic and parasympathetic activity). However, these models demonstrated a much lower fitting with lower R^2^ values (R^2^ for LF 0.37±0.17, Ptot 0.53±0.23 and LF/HF 0.36±0.19), contrasting with the R^2^ value of 0.84 for HF. It was noted that there was no significant correlation between measured and estimated performances and HF indices when temporal indices of heart rate variability were used. This indicated that these last measurements of ANS activity were less accurate for measuring heart rate variability [25].

### Gain and Decay Parameters

Gain parameters and time constants of decay belonging to the positive and negative parts of the modeling equation were assessed. The mean values of the gain parameters (k_1_ and k_2_), calculated from measured and estimated performance fitting were 0.00007 (SD 0.00006) and 0.0011 (SD 0.0014) respectively. The mean values of the gain parameters (k_1_ and k_2_), calculated from measured and estimated HF fitting were 0.007 (SD 0.006) and 0.18 (SD 0.16) respectively (Table 2). The mean values of the time constants of decay (τ_1_ and τ_2_), calculated from measured and estimated performance fitting were 50 days (SD 14) and 4.8 days (SD 2.9) respectively. The mean values of the time constants of decay (τ_1_ and τ_2_), calculated from measured and estimated HF fitting were 42 days (SD 15, Table 2) and 4.2 days, respectively (SD 2.3, Table 2).

**Table 2:** Model parameters (mean±SD) for performance and HF spectral components of HRV.

	Performance							HF spectral component of HRV						
	Initial level	k_1_	k_2_	τ_1_(days)	τ_2_(days)	t_n_P_ (days)	t_g_P_ (days)	Initial level	k_1_	k_2_	τ_1_ (days)	τ_2_ (days)	t_n_HF_(days)	t_g_HF_(days)
**Mean**	1.42	0.00007	0.0011	50	4.8	14.47	25.87	8.36	0.007	0.18	42	4.2	12.71	22.93
**SD**	0.12	0.00006	0.0014	14	2.9	4.99	9.17	5.22	0.006	0.16	15	2.3	4.66	8.90

**Initial level**: Initial basic level of performance and HF spectral component of HRV**.**

**k_1_** and **k_2_** : multiplying factors respectively for the positive and negative component of performance and HF spectral component of HRV. **τ_1_** and **τ_2_** : time constants of decay for positive and negative components of performance and HF spectral component of HRV.

**t_n_** : Critical period pre-competition in which training has a negative effect on performance (t_n_P_) and HF spectral component of HRV (t_n_HF_).

**t_g_** : time pre-competition when training has maximum benefit.

**k_1_** and **k_2_** are expressed in arbitrary units depending on units used to quantify training load, performance and HF spectral component of HRV.

### Time to Recover and Time to Peak Performance

**Figure 3:**
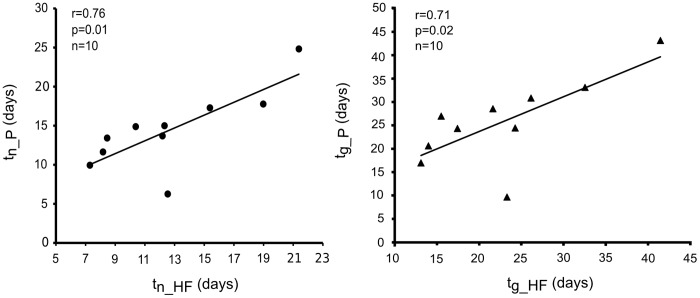
Correlation between recovery time for performance (t_n_P_) and HF power (t_n_HF_) and between peak time for performance (t_g_P_) and HF power (t_g_HF_). Significant correlation was demonstrated first, between the time to recover to the initial level of performance (t_n_P_) and HF power (left panel) and second, between the time to peak performance and HF power (t_g_HF_).

There was a significant relationship between the time to recover performance after a training bout (t_n_P_) and the time to recover HF (t_n_HF_) (r = 0.76, p = 0.01, Figure 3 left Panel). It was noted that t_n_P_ and t_n_HF_ showed large variations between swimmers with a range of 6–24 days and 8–21 days respectively. There was also a significant relationship between the time to peak performance after a training bout (t_g_P_) and the time to peak HF (t_g_HF_) (r = 0.71, p = 0.02, Figure 3, right Panel). The parameters t_g_P_ and t_g_HF_ showed large variations between swimmers with a range of 9–43 days and 13–41 days respectively. Only one swimmer did not fit the relationship for t_n_ nor t_g._ It is noted that this subject was a specialized sprinter not an endurance swimmer.

### Positive and Negative Influences

**Figure 4:**
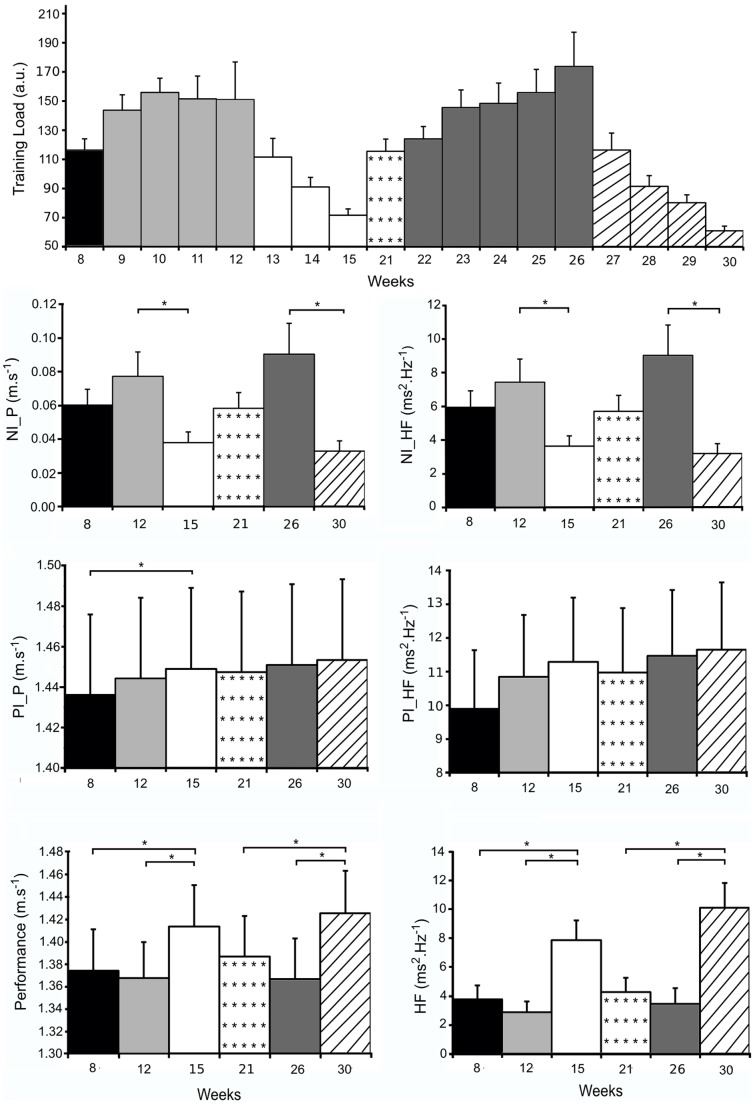
Values of training load, NI_P, PI_P, Performance, NI_HF, PI_HF, and HF power for the whole group. Results recorded in the week before beginning IT periods (W8 and W21); the last week of IT periods (W12 and W26); and taper (W15 and W30).

Positive and negative influences derived from the model are shown in Figure 2 for swimmer #4 and in Figure 4 for the whole group. The positive influence increases slowly then remains at a high level without being disrupted by the two high intensity training periods, then shows a further increase during the two recovery periods. This is observed for PI_P (Figure 2, panel E, Figure 4) and for PI_HF (Figure 2 panel F, Figure 4), while the later increases slightly more slowly than the former. It is possible that the further increase in positive influences observed for the second training cycle may be related to the additional one week recovery period of that cycle. Conversely, both NI_P and NI_HF increased during each high intensity training period and then decreased during the subsequent recovery period. The variations in negative influences are quicker than the respective positive influences. This results in the high intensity training periods having had a negative impact on both performance and HF. However, this negative influence of training is only transient because the reduction of training amounts during the recovery period allows the negative influence to decrease, and thus performance and HF to increase.

### Relationship between HF Power and RR Interval Length

**Figure 5:**
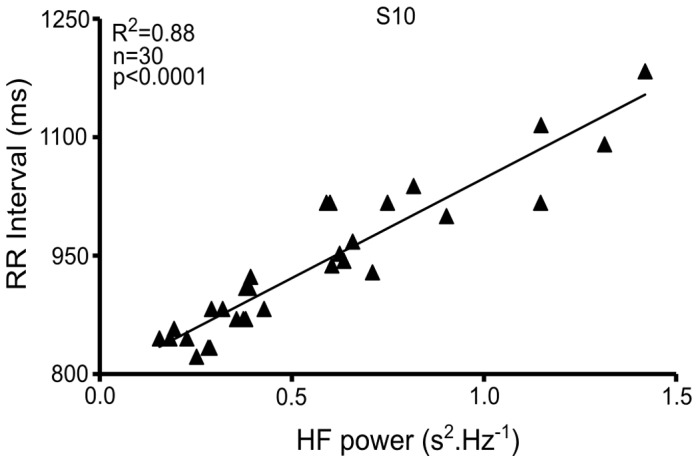
Representation of the relationship between RR interval and HF power for swimmer #10.

The minimal and maximal length of the RR interval and also the minimal and maximal HF power value was investigated for each subject of the study (Table 3). There is a linear increase in RR interval length with the increasing HF power of HRV (Figure 5) for eight subjects of the study (R^2^ from 0.49 to 0.88, p<0.001; S1: 0.49; S2: 0.55; S3: 0.80; S4: 0.81; S5: 0.72; S6: 0.76; S7: 0.74; S10: 0.88). One subject presented a weaker relationship characterized by R^2^ = 0.27 (S8) and another (S9) presented no relationship (R^2^<0.001).

**Table 3:** Values of RR interval length (ms) and HF power (s^2^.Hz^−1^).

	RR length (ms)			HF power (s^2^.Hz^−1^)		
Subjects	RR length min	RR length max	RR length mean	HF min	HF max	HF mean
**S1**	909.09	1132.08	1025.49±75.00	2.034	17.528	8.378±3.355
**S2**	923.08	1304.35	1085.54±101.58	4.527	18.357	10.316±2.911
**S3**	853.12	1363.64	1053.09±136	0.864	9.611	3.385±2.402
**S4**	800.00	1030.93	874.92±52.51	2.572	11.268	5.587±2.269
**S5**	796.07	1088.14	903.47±86.80	0.296	4.886	1.137±0.9429
**S6**	866.43	1090.91	958.48±66.40	0.109	5.164	2.153±1.472
**S7**	1034.48	1224.43	1126.43±45.30	1.930	9.430	4.841±1.864
**S8**	869.56	1153.85	1030.98±71.61	1.669	9.611	4.270±1.914
**S9**	1077.20	1388.89	1220.41±69.50	5.807	13.671	8.833±2.562
**S10**	821.92	1183.43	936.77±92.48	0.155	1.419	0.560±0.343

## Discussion

Using the model of Banister et al. [1] for the analysis of elite swimmers over a full season under standard training conditions a clear relationship between performance and parasympathetic activity was demonstrated (Figure 1).

### Model Statistics and Parameters

Our study demonstrated a strong relationship between performance and HF power for each subject. The study duration allowed calculation of the logarithmic-shaped fit for HF power and performance. This is in contrast to previous studies on swimmers [5], runners [4] and workers [26]. These latter studies were carried out over shorter periods and demonstrated linear relationships [5]. The choice of an aerobic high intensity exercise probably participated in the improved quality of the model fit [27].

The similarity between performance and the HF responses to training was demonstrated through the adequacy of the two antagonistic component model proposed by Banister et al. [1]. This provides a good representation of the variations of both variables used for systems output. In spite of the importance of inter-subject variability, the estimates of the time constants were similar considering the fit of performance or that of the HF spectral component of HRV (50±14 *vs.* 42±15 days for τ_1_ and 5±3 *vs*. 4±2 days for τ_2_ respectively). Previous application of the two-component model in elite swimmers [16] showed close estimates for τ_1_ (41±13 days). The estimates of τ_2_ (12±7 days) in this previous study were however higher than the values obtained in our study for both performance and HF power. This difference in τ_2_ could be explained by our study population being younger and less trained than older and more experienced elite athletes.

To go beyond the comparison of model parameters we computed t_n_ and t_g_ from the time constant and gain term of the two components. It has been suggested that the optimal duration of taper to maximize performance would be between t_n_ and t_g_ [18]. This is because the taper needs to be long enough to dissipate fatigue from past training but not too long to avoid the loss of adaptations. Previous models applied to elite swimmers [16] gave an optimal duration of between two (t_n_) and four weeks (t_g_) in accordance with the review of the literature [9], [28]. The present study concurs with these estimates for performance (t_n_P_ and t_g_P_) and HF power (t_n_HF_ and t_g_HF_).

A similar impact of training on both performance and HF power was evidenced by the correlation observed between t_n_P_ and t_n_HF_ and between t_g_P_ and t_g_HF_ (Figure 4). These findings demonstrate that the HF spectral component of HRV could be a useful tool if used systematically to optimize training load throughout a competition season.

### Intensive Training Periods and Taper

Improvements in performance, as well as in HF power, during the two taper periods could be related to recovery. This is particularly related with a significant decrease in the negative influence in response to the reduced training amount [16], [21]. As a result, negative influence appears to be the major determining parameter for both performance [16] and HF power. Conversely, positive influence did not present significant variations during the two taper periods for either performance or HF power and remained constantly high during the two periods. Before the two tapers, even if the level of positive influences was high enough to achieve a high performance without tapering, the level of negative influences prevented the swimmers from maximizing their HF power level and performing at their best. This suggests that optimal taper would depend on the characteristics of the prior intensive training period [20], [21].

According to the fatigue and adaptation model, fatigue increases more than adaptation in response to a training bout. When the training load is reduced, fatigue dissipates faster than adaptation, allowing peak performance. This suggests that a longer period would be required to recover from ‘overload’ training and HF power would appear useful for ensuring that, at exactly the right moment, the taper reduced the negative influences while keeping positive influences at a consistently high level.

We believe that HF power could be used to determine an individual’s optimal training load during both the intensive training and taper periods. A decreased HF power could reflect an insufficient recovery effect from previous training periods which may indicate unfavorable conditions for performance improvements. An important increase in HF power during an intensive training period may suggest insufficient training intensity.

Several cross-sectional and longitudinal studies have highlighted an association between endurance training and cardiac parasympathetic outflow. Garet et al. [5] demonstrated that the improvement in swimming performance observed after a taper period was correlated with the concomitant HF rebound. Kiviniemi et al. [6] demonstrated the VO_2_peak increase induced by a training program to be improved by transitory decreases in training amount each time HF power decreases below a threshold. Both experiments suggest that decreased HF power could have been due to increased negative influences. In the present study, a possible saturation phenomenon of HF power was taken into account in the analysis of vagal function, through the analysis of the relationship between RR interval and HF power. Instead of the linear increase in RR interval observed with increasing vagal tone, previous studies [29], [30] have reported a plateau or even a decrease of HF power until HR reached ∼50 beats per minute. This phenomenon, referred to as saturation of HRV, should reflect the inability of HRV measures to detect changes in cardiac vagal outflow at low HR levels. In our study, HF power increased linearly as the RR interval increased without a plateau or even a decrease of HF power, for nine subjects. Only one subject did not present a significant linear relationship between HF power and HR.

### Mechanisms Underlying the Relationship between Performance and Cardiac Vagal Outflow

The correlations between HF power and performance raise the question of the mechanisms underlying the relationship between nocturnal vagal activity and the training response. Previous studies have demonstrated the coherence of the model as a method to describe biological responses to training. The variations in several serum enzyme activities in runner [31] and hormonal adaptations in six elite weightlifters [15], [32] have been shown to significantly correlate with modeled fatigue and adaptation indices. The fatigue and adaptation model shows that the variations in performance and HF power with intensive training and taper periods are correlated to the variations in the negative influences of training. The variation in HF power could be one index of the fatigue status of an athlete. The strong relationship observed between the time courses of these two variables suggests however that parasympathetic activity could have a direct bearing on performance. Hedelin et al. [33] demonstrated that the ability to further improve VO_2_max with training in aerobically fit subjects was related to a higher HF power. This was confirmed by Hautala et al. [34] who observed the HF power recorded nocturnally to be a powerful independent predictor of the response to an aerobic training program. This could suggest that higher parasympathetic activity was a cause rather than an effect of an increase in aerobic fitness. There is however no data to support a cause-effect relationship. Hautala et al. [34] hypothesized that a genetically determined common denominator could partially explain adaptations to aerobic training and HR variability. This remains to be investigated.

Our results demonstrated a high fit between the levels of performance and the indices of parasympathetic activity in response to training. This model fit is highly individual and provides a means to describe precisely the displacement along these individual curves in response to the antagonists of training and recovery periods.

### Conclusion

This study demonstrated two important findings. First, a strong correlation between performance and HF spectral component of HRV in each subject; and second, that the two-component systems model originally proposed to explain the variations in performance with intensive training is also adequate for describing changes in HRV. Using performance or HF as the systems output provided the same information on the impact of training on the fatigue and adaptation status of the athlete. Our results demonstrated the relevance of change in HRV as a valuable tool to assess the physiological training-induced responses and to further optimize athletic performance. Furthermore, the HRV spectral component of HRV could also serve as an alternative to performance for mathematical modeling of training effects. The advantage is the greater number of possible measurements without interfering with the training schedule, especially in sporting activities in which performance cannot be readily measured.

## Supporting Information

Appendix S1 **Wavelet transform.**
(DOC)

Appendix S2 **Quantification of the training amount.**
(DOC)

Appendix S3 **Mathematical modeling of the responses to training.**
(DOC)

## References

[1] Banister E, Calvert T, Savage M, Bach T (1975) A systems model of training for athletic performance. Australian Journal of Sports Medicine 7: 57–61.

[2] Kenney WL (1988) Endurance training increases vagal control heart rate. In: Dotson CO, Humphrey JH, editors. Exercise physiology: current selected research. New York: AMS Press. 59–65.

[3] Furlan R, Piazza S, Dell’Orto S, Gentile E, Cerutti S, et al (1993) Early and late effects of exercise and athletic training on neural mechanismes controlling heart rate. Cardiovascular research 27: 482–488. 8490949 10.1093/cvr/27.3.482

[4] Pichot V, Roche F, Gaspoz J, Enjolras F, Antoniadis A, et al (2000) Relation between heart rate variability and training load in middle- distance runners. Medicine and Science in Sports and Exercise 32: 1729–1736. 11039645 10.1097/00005768-200010000-00011

[5] Garet M, Tournaire N, Roche F, Laurent R, Lacour J, et al (2004) Individual interdependence between nocturnal ANS activity and performance in swimmers. Medicine and Science in Sports and Exercise 36: 2112–2118. 15570148 10.1249/01.mss.0000147588.28955.48

[6] Kiviniemi A, Hautala A, Kinnunen H, Tulppo M (2007) Endurance training guided individually by daily heart rate variability measurements. European Journal of Applied Physiology 101: 743–751. 17849143 10.1007/s00421-007-0552-2

[7] Wenger H, Bell G (1986) The interactions of intensity, frequency and duration of exercise training in altering cardiorespiratory fitness. Sports Medicine 3: 346–356. 3529283 10.2165/00007256-198603050-00004

[8] Fry R, Morton A, Keast D (1992) Periodisation and the prevention of overtraining. Canadian Journal of Sport Sciences 17: 241–248. 1325265

[9] Mujika I, Padilla S (2003) Scientific bases for precompetition tapering strategies. Medicine and Science in Sports and Exercise 35: 1182–1187. 12840640 10.1249/01.MSS.0000074448.73931.11

[10] Busso T, Benoit H, Bonnefoy R, Feasson L, Lacour J (2002) Effects of training frequency on the dynamics of performance response to a single training bout. Journal of Applied Physiology 92: 572–580. 11796666 10.1152/japplphysiol.00429.2001

[11] Kuipers H, Keizer HA (1988) Overtraining in elite athletes. Review and directions for the future. Sports Med 6: 79–92. 3062735 10.2165/00007256-198806020-00003

[12] Fry R, Morton A, Garcia-Webb P, Crawford G, Keast D (1992) Biological responses to overload training in endurance sports. European Journal of Applied Physiology 64: 335–344. 10.1007/BF00636221 1592059

[13] Banister E, Hamilton C (1985) Variations in iron status with fatigue modelled from training in female distance runners. European Journal of Applied Physiology 54: 16–23. 10.1007/BF00426292 4018049

[14] Busso T, Carasso C, Lacour J (1991) Adequacy of a systems structure in the modeling of training effects on performance. Journal of Applied Physiology 71: 2044–2049. 1761506 10.1152/jappl.1991.71.5.2044

[15] Busso T, Hakkinen K, Pakarinen A, Kauhanen H, Komi PV, et al (1992) Hormonal adaptations and modelled responses in elite weightlifters during 6 weeks of training. Eur J Appl Physiol Occup Physiol 64: 381–386. 1592066 10.1007/BF00636228

[16] Mujika I, Busso T, Lacoste L, Barale F, Geyssant A, et al (1996) Modeled responses to training and taper in competitive swimmers. Med Sci Sports Exerc 28: 251–258. 8775162 10.1097/00005768-199602000-00015

[17] Busso T (2003) Variable dose-response relationship between exercise training and performance. Medicine and Science in Sports and Exercise 35: 1188–1195. 12840641 10.1249/01.MSS.0000074465.13621.37

[18] Fitz-Clarke JR, Morton AR, Banister EW (1991) Optimizing athletic performance by influence curves. Journal of Applied Physiology 71: 1151–1158. 1757312 10.1152/jappl.1991.71.3.1151

[19] Mujika I (1998) The influence of training characteristics and tapering on the adaptation in highly trained individuals: a review. International Journal of Sports Medicine 19: 439–446. 9839839 10.1055/s-2007-971942

[20] Thomas L, Busso T (2005) A theoretical study of taper characteristics to optimize performance. Medicine and Science in Sports and Exercise 37: 1615–1621. 16177616 10.1249/01.mss.0000177461.94156.4b

[21] Thomas L, Mujika I, Busso T (2008) A model study of optimal training reduction during pre-event taper in elite swimmers. Journal of Sports Sciences 26: 643–652. 18344135 10.1080/02640410701716782

[22] Thomas L, Mujika I, Busso T (2009) Computer simulations assessing the potential performance benefit of a final increase in training during pre-event taper. Journal of Strenght and Conditioning Research 23: 1729–1736. 10.1519/JSC.0b013e3181b3dfa1 19675490

[23] Calvert T, Banister E, Savage M, Bach F (1976) A Systems Model of the Effects of Training on Physical Performance. IEEE Trans Syst Man Cybern 6: 94–102.

[24] Jensen-Urstad K, Saltin B, Ericson M, Storck N, Jensen-Urstad M (1997) Pronounced resting bradycardia in male elite runners is associated with high heart rate variability. Scandinavian Journal of Medicine and Science in Sports 7: 274–278. 9338944 10.1111/j.1600-0838.1997.tb00152.x

[25] Pichot V, Gaspoz J, Molliex S, Antoniadis A, Busso T, et al (1999) Wavelet transform to quantify heart rate variability and to assess its instantaneous changes. Journal of Applied Physiology 86: 1081–1091. 10066727 10.1152/jappl.1999.86.3.1081

[26] Pichot V, Bourin E, Roche F, Garet M, Gaspoz JM, et al (2002) Quantification of cumulated physical fatigue at the workplace. Pflugers Arch-European Journal of Physiology 445: 267–272. 12457247 10.1007/s00424-002-0917-7

[27] Casey A, Mann R, Banister K, Fox J, Morris P, et al (2000) Effect of carbohydrate ingestion on glycogen resynthesis in human liver and skeletal muscle, measured by 13C MRS. American journal of Physiology Endocrinology and metabolism 278: E65–E75. 10644538 10.1152/ajpendo.2000.278.1.E65

[28] Bosquet L, Montpetit J, Arvisais D, Mujika I (2007) Effects of Tapering on Performance: A Meta-Analysis. Medicine and Science in Sports and Exercise 39: 1358–1365. 17762369 10.1249/mss.0b013e31806010e0

[29] Goldberger J, Challapalli S, Tung R, Parker M, Kadish A (2001) Relationship of heart rate variability to parasympathetic effect. Circulation 103: 1977–1983. 11306527 10.1161/01.cir.103.15.1977

[30] Tulppo M, Mäkikallio T, Seppänen T, Airaksinen J, Huikuri H (1998) Heart rate dynamics during accentuated sympathovagal interaction. American Journal of Physiology Heart and Circulatory Physiology 274: H810–H816. 10.1152/ajpheart.1998.274.3.H810 9530192

[31] Banister E (1982) Modeling Elite Athletic Performance In: MacDougall J, Wenger H, Green H, editors. Physiological Testing of the High-Performance Athlete. Champaign, IL: Human Kinetics.

[32] Busso T, Häkkinen K, Pakarinen A, Carasso C, Lacour J, et al (1990) A systems model of training responses and its relationship to hormonal responses in elite weight-lifters. European Journal of Applied Physiology 61: 48–54. 10.1007/BF00236693 2289497

[33] Hedelin R, Bjerle P, Henriksson-Larsen K (2001) Heart rate variability in athletes: relationship with central and peripheral performance. Medicine and Science in Sports and Exercise 33: 1394–1398. 11474344 10.1097/00005768-200108000-00023

[34] Hautala A, Mäkikallio T, Kiviniemi A, Laukkanen R, Nissilä S, et al (2003) Cardiovascular autonomic function correlates with the response to aerobic training in healthy sedentary subjects. American Journal of Physiology Heart and Circulatory Physiology 285: H1747–H1752. 12816748 10.1152/ajpheart.00202.2003

