# Supplementary material for: A Model for the Training Effects in Swimming Demonstrates a Strong Relationship between Parasympathetic Activity, Performance and Index of Fatigue
Source: PLoS One. 2012 Dec 20;7(12):e52636. doi: 10.1371/journal.pone.0052636 (PMC3527593; doi:10.1371/journal.pone.0052636)
Supplement: Appendix S1 — Wavelet transform. (DOC) [file pone.0052636.s001.doc]

***Appendix 1:* Wavelet transform**

The decomposition of the RR list by Wavelet transform requires a ψ function adequately regular and localized, named the Mother function. Starting from this initial function, a family of functions is built by dilatation and translocation to constitute the so-called Wavelet frame. The analysis amounts to sliding a window of different weights (corresponding to different levels) containing the Wavelet function, along the whole signal. In our analysis, we chose the Daubechies 4 Wavelet transform. For each record, the Wavelet coefficients were calculated on sets of 256 RR intervals, giving seven separate levels of analysis named 2, 4, 8 ... 128. We then calculated the variability power, level by level, as the sum of the squares of the coefficients.

Wavelet indices include the sum of Wavelet power coefficients at levels 2, 4 and 8, which provide an index of parasympathetic activity; Wavelet power coefficients at levels 16 and 32 (LFWavelet) roughly represent both parasympathetic and sympathetic activities; Wavelet power coefficients at levels 64 and 128 (VLFWavelet) roughly represent an index of sympathetic activity; and the LFWavelet/HFWavelet ratio provides an evaluation of the autonomic nervous system balance (sympathetic/parasympathetic). Total frequency power was also calculated (PtotWavelet).
