# Supplementary material for: A Model for the Training Effects in Swimming Demonstrates a Strong Relationship between Parasympathetic Activity, Performance and Index of Fatigue
Source: PLoS One. 2012 Dec 20;7(12):e52636. doi: 10.1371/journal.pone.0052636 (PMC3527593; doi:10.1371/journal.pone.0052636)
Supplement: Appendix S2 — Quantification of the training amount. (DOC) [file pone.0052636.s002.doc]

***Appendix 2:* Quantification of the training stimulus**

1) Pool workouts: Each athlete performed a 400 m freestyle swim time-trial at maximal intensity and then a 1500 m freestyle swim to obtain indicators of the maximal aerobic speed and the speed at which the onset of blood lactate accumulation (OBLA) occurs, respectively. From each individual’s results for these two tests, all the training performed in the water was then divided into seven intensity levels. Intensities I and II represented swimming speeds inferior to that for the onset of blood lactate accumulation. Intensities III and IV represented swimming speeds equal and slightly above OBLA, respectively. Intensity V represented the maximal aerobic speed. High intensity swimming causing blood lactate accumulation was defined as intensity VI and maximal intensity sprint swimming as intensity VII. The distance swum at each training intensity was associated with a coefficient calculated using Banister’s method [31].

$$y=0.64e^{1.92x} (male)$$

$$y=0.86e^{1.67x} (female)$$

where y=multiplying factor, x=delta HR ratio during exercise, and e is the Napieran logarithm.

2) Dry land workouts: equivalent intensity ratings were estimated for the different types of dry land workout in order to quantify the water equivalents of the dry land training. Using Mujika’s method [16], it was taken that a one-hour session on land was equivalent to two kilometres of swimming with the hour composed of 30 minutes of low intensity warm up and stretching exercises (equivalent intensity II), 15 minutes of submaximal strength exercises (equivalent intensity III) and 15 minutes of maximal strength exercise (equivalent intensity V).

3) The total weekly training load: W (measured in arbitrary units) (equation 1), was computed as the sum of kilometres (Z1, Z2, Z3…Z7) swum at each training intensity multiplied by first their HR delta ratio (Δ1, Δ2, Δ3…Δ7) and then their respective coefficients (k1, k2, k3…k7), added to the water equivalent of the dry land training.

$$W=(Z1*\Delta1*k1)+(Z2*\Delta2*k2)+(Z3*\Delta3*k3)+...+(Z7*\Delta7*k7)+Dry land training$$
