# Supplementary material for: A Model for the Training Effects in Swimming Demonstrates a Strong Relationship between Parasympathetic Activity, Performance and Index of Fatigue
Source: PLoS One. 2012 Dec 20;7(12):e52636. doi: 10.1371/journal.pone.0052636 (PMC3527593; doi:10.1371/journal.pone.0052636)
Supplement: Appendix S3 — Mathematical modeling of the responses to training. (DOC) [file pone.0052636.s003.doc]

***Appendix 3:* Mathematical modeling of the responses to training**

In this study, the use of systems modeling to describe adaptations to training consists in relating both performance and the HF spectral component of HRV (system output) to the amount of training (system input).

*Two-component model.* The model, initially proposed by Banister et al. [1], is a two-component model characterized by two gain terms (k_1_ and k_2_), and two time constants (τ_1_ and τ_2_).

$$\hat{p}^{n}=p^{*}+k_{1}\sum_{i=1}^{n-1} w^{i}e^{-(n-i)/\tau1}-k_{2}\sum_{i=1}^{n-1} w^{i}e^{-(n-i)/\tau2}$$

The calculated performance noted p(t) is obtained by convolution product of the training dose w(t) with the impulse response added to basic level of performance noted p*. W(t) is considered to be a discrete function, i.e. a series of impulses each day, w^i^ on day i, the convolution product becomes a summation in which modeled performance, p^n^ on day n, is estimated by mathematical recursion from the series of w^i^. The calculated level of HF power was obtained following exactly the same method as described above. The parameters for each model were determined by fitting the estimated performance and HF power to the measured performance and HF power by the least square method. All the model parameters were determined minimizing the Residual Sum of Squares (RSS) between estimated and measured performance and the HF spectral component of HRV.

$$RSS=\sum_{n=1}^{N} \left[p^{n}-\hat{p}^{n}\right]^{2}$$

where n takes the N values corresponding to the days of measurement of the actual performance.

The time response of performance and HF power to a single training session was characterized by variables derived from model parameters. The model proposed by Banister et al*.* [1] assumes that, in response to a training impulse, performance first decreases and then returns to the initial level after a time t_n_ and then peaks at a higher level after a time t_g_ as follows:

$$t_{n}=\frac{ \tau1 \tau2 }{\tau1-\tau2} \ln\left(\frac{k_{2}}{k_{1}}\right) and t_{g}=\frac{ \tau1 \tau2}{\tau1 - \tau2} \ln\left(\frac{\tau1 k2}{\tau2 k2}\right)$$

Accordingly, we computed the time to recover performance levels (tn_P) and HF power (tn_HF), and the time to peak performance (tg_P) and HF power (tg_HF). The negative and the positive functions of the model have been associated with fatigue and fitness, respectively [13,31,32]. Fatigue and fitness indicators have also been computed from the combined effects of both model functions on performance [15]. These influence curves have been described in detail to determine the training program that would give the best performance at a target time [18]. The influence of a training stimulus imposed at day i on performance and on HF power at day n is calculated from the following equation:

$$E(i/n)=k_{1}w^{i}e^{-(n-i)/\tau1}-k_{2}w^{i}e^{-(n-i)/\tau2}$$

The negative influence of training was distinguished from the positive influence for the purpose of building fatigue and fitness profiles. The initial decrease in performance and HF power after completion of training was considered as fatigue. Conversely, the improvement in performance obtained a few days later was considered as a gain in fitness. The values of negative influence and positive influence on performance (NI_P and PI_P) and on HF power (NI_HF and PI_HF) on day n were estimated from the sum of influences of each past training amount depending on whether the result was negative or positive.

$$NI^{n}=\sum_{i=1}^{n-1} \left|E(i/n)\right|, when E(i/n)<0$$

$$PI^{n}=p^{*}+\sum_{i=1}^{n-1} \left|E(i/n)\right|, when E(i/n)>0$$
